# Factors associated with Schistosomiasis outbreak at Omindamba primary school, Omusati region, Namibia: a case-control study, March 2016

**DOI:** 10.11604/pamj.2017.28.212.11458

**Published:** 2017-11-07

**Authors:** Uzenia Ndatelela Mupakeleni, Kofi Mensah Nyarko, Francina Ananias, Peter Nsubuga, Emmy-Else Ndevaetela

**Affiliations:** 1 Namibia Field Epidemiology and Laboratory Training Programme, School of Public Health, University of Namibia, Windhoek, Namibia; 2 Ministry of Health and Social Services, Omusati Region, Namibia; 3 Global Public Health Solutions, Atlanta, GA, USA; 4 Ministry of Health and Social Services, Epidemiology Division, Namibia

**Keywords:** Schistosomiasis outbreak, Schistosoma hematobium, Omusati region, Namibia, case-control study

## Abstract

**Introduction:**

On march 2, 2016, the principal of Omindamba primary school in Outapi district notified the Outapi hospital of a cluster of students complaining of dysuria and passing bloody urine. We conducted an investigation to identify the agent, source of infection, and to determine factors associated with the outbreak.

**Methods:**

An unmatched 1:1 case-control study was conducted. A case was defined as any student of Omindamba primary school, who passed bloody urine with or without dysuria or lower abdominal pains from 2^nd^- 4^th^ March 2016, during the time of the investigation. A control was a classmate of a case. We collected demographic, clinical and environmental data.

**Results:**

125 cases and 125 controls were enrolled into the study. The mean age for cases was 11.3 years and controls was 11.0 years, with standard deviation of 3.2 years in both cases and controls. The most affected age group was 11-15 years with 63 (50.4%) cases, followed by 6-10 years with 51(40.8%) cases. *Schistosoma hematobium* was isolated in the urine specimens examined. Swimming in the canal (adjusted OR = 3.58; 95% CI = 1.14-11.2; p-value = 0.03), and using Etaka pond as a source of water (adjusted OR = 1.95; 95% CI = 1.09-3.50, p-value = 0.02) were independent factors associated with contracting schistosomiasis.

**Conclusion:**

The Schistosomiasis outbreak among the school children was caused by *Schistosoma hematobium*. Swimming in the canal and using Etaka pond as a source of drinking water were the predictive factors for the infection. A community-based health education on the prevention of Schistosomiasis was implemented.

## Introduction

Schistosomiasis is an acute and chronic neglected ancient human disease caused by parasitic worms, affecting people worldwide, particularly in the poorest communities [1]. People are infected during routine agricultural, domestic, occupational and recreational activities which expose them to infested water [2]. When an infected person defecates, or urinates in or near freshwater, releasing a proportion of the eggs into the water, eggs hatch and infect their intermediate snail host. The eggs hatch to release miracidia that then infect the snails. The miracidia develops within the snails into cercariae that are then released by the infected snails to penetrate a person's skin and develop into mature worms [3]. Lack of hygiene and certain play habits of school-aged children such as swimming or fishing in infested water make them especially vulnerable to infection [1]. All ages are at risk of infection with freshwater exposure in endemic areas [4]. Environmental, climatic and demographic changes, as well as increasing urbanization, may have a considerable effect on the transmission of this infectious diseases [5]. There are two types of schistosomiasis disease-urinary and intestinal schistosomiasis. Urinary schistosomiasis is caused by *S. haematobium,* while intestinal schistosomiasis is caused by either *S. mansoni, S. japonicas, S. intercalantum or S. mekongi* [6]. *Schistosoma* parasites can penetrate the skin of persons who come in contact with contaminated freshwater, typically when wading, swimming, bathing, or washing. Schistosomiasis affects almost 300 million people worldwide each year. More than 61.6 million people were reported to have been treated for schistosomiasis in 2014 [7]. A total of 258 million people was estimated to require treatment for Schistosomiasis in 2014; of whom 123 million (47.6%) were school-age children (5-14 years of age). Additionally, 91.4% of the people estimated to require treatment for schistosomiasis lived in the African region [7]. Schistosomiasis causes severe morbidity in large parts of Africa, Asia, and South America. The World Health Organization (WHO) recommends preventive chemotherapy and transmission control (PCT) with praziquantel for the prevention, control, and treatment of the disease, which is provided to target populations through Mass Drug Administration (MDA), i.e., the treatment of entire populations irrespective of disease status [8]. In Namibia, the disease is endemic in the northern regions of the country, especially in Kavango and Omusati regions [9]. The average of 27 schistosomiasis cases are reported in the region per month [10]. According to Namibia Statistic Agency (NSA) in 2011, 51.6% of the households in Omusati region had safe running water supplies (from the taps) inside or outside their dwellings, for cooking and drinking, while 48.4% had unsafe water supplies, either from the river, dam or streams [11]. On March 2, 2016, the principal of Omindamba Primary School in Outapi district notified the Outapi hospital of a cluster of students complaining of dysuria and passing bloody urine. As a public health action, a team from Outapi district consisting of a surveillance focal person, environmental health officials and Namibia Field Epidemiology and Laboratory Training Program (NamFELTP) resident went to the school to investigate the outbreak. The investigation aimed to identify the agent, source of infection, and to determine factors associated with the outbreak at Omindamba primary school.

## Methods

**Study setting:** Omusati region is one of the Northern regions of Namibia, with an area of 26,551 km^2^, bordering the Cunene Province of Angola on the north, Ohangwena region, Namibia on the northeast, Oshana region, Namibia to the east and Kunene region, Namibia to the south and west. Omusati region has a canal which carries water from the Ruacana River to Oshakati, passing through Outapi. Water from this canal has been used to irrigate a large, government-run farm at Etunda where crops such as maize, watermelon, and bananas are grown. Community members use water from the canal for domestic use and agricultural purposes. There is a big pond (Etaka) and other several small ponds in the region, where community members swim and collect water for domestic use. Omindamba Primary School has a total number of 468 students, with 262 males and 206 females.

**Study design:** We conducted an unmatched 1:1 case-control study among pupils attending at Omindamba Primary School.

**Case definitions:** A case was defined as any student of Omindamba primary school, who passed bloody urine with or without dysuria or lower abdominal pains from 2^nd^ March, 2016 to 4^th^ March, 2016. A control was a classmate of a case, who had no dysuria; no lower abdominal pain and did not pass bloody urine at the equivalent time of the investigation.

**Selection of cases and controls:** After health education was given on prevention, treatment and complications; students were encouraged to be free and voluntary report themselves to the investigators. For the lower grade students (grade 1-3), teachers were advised to identify students who visit toilets frequently and to be interviewed before regarded as cases. We visited class-by-class to identify cases. Controls were randomly selected from the same class as cases. We used a simple random sampling method to select controls. Cases randomly picked a paper from a box which contained the names of all students who were not cases and then we interviewed them as possible controls.

**Inclusion and exclusion criteria:** We recruited all the cases (125) in the study and the randomly selected 125 controls. Students who were recently treated for the similar signs and symptoms were excluded in the study. All the cases were treated with Praziquantel as per Namibian treatment guidelines, except those who had been treated recently with Praziquantel. No control that was selected had received Praziquantel recently. We interviewed all the female cases on their last normal menstrual periods to rule out pregnancies before administered Praziquantel [**12**].

**Data collection:** We met with the school principal and teachers; and gathered students in association with a reported cluster of students complaining of dysuria and hematuria. Five urine specimens were collected from randomly selected students who had dysuria and sent to Namibia Institute of Pathology (NIP) for analysis. *Schistosoma hematobium* ova were identified in the urine samples using microscopic method. Five millilitres of urine were centrifuged in a test tube at 1800-2000rpm for 5 minutes. The supernatant was decanted and the deposit re-suspended. A wet preparation was made with a drop on the slide and covered with a cover slip. The wet preparation was examined microscopically using 10x objective and 40X objective. Ova of *Schistosoma hematobium* was then observed. We developed a line list using the case definition to record all students who qualify as cases. After the laboratory confirmation of schistosomiasis, we adjusted the line list by adding the possible risk factors of the disease. Variables contained in the line list were demographic, clinical and environmental data. Demographic data were age, sex, residential address, employment status of the caretakers. Clinical data in the line list included the dates signs and symptoms started; and environmental information included the source of drinking water, whether they swim in canal water or not, and whether they consume the ground water, if so, is it treated or boiled before use. Data were collected using an interviewer-administered checklist for the study participants. We then entered it into a computer based line listing form.

**Data management:** We cleaned and analyzed data with Epi Info™ version 7(US Centers for Disease Control and Prevention, 2011) and Microsoft Excel (Microsoft Corp., Seattle). Frequencies, means, and proportions were generated; and bivariate and logistic regression analyses were performed to generate Odds Ratios, 95% confidence intervals, and p-values. Statistical significance was determined at p-value ≤ 0.05.

**Ethical considerations:** Outbreak response is part of public health emergency; therefore, no ethical approval is needed, but rather administrative approval was sought form the Ministry of Health. Permissions to carry out the investigation were also sought from the Ministry of Health and Social Services-Omusati region, the school principal, students, and their respective teachers. Verbal informed consent was sought from the students. Confidentiality was maintained throughout and after the study. Names of the students were not included on the checklist for confidentiality reasons.

## Results

**Figure 1:**
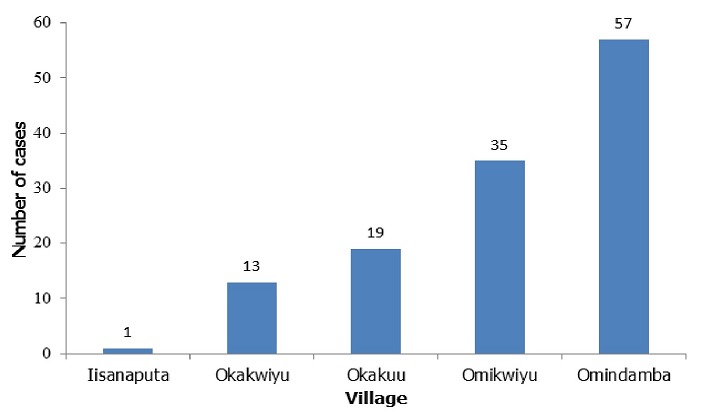
Geographical distribution of cases of Omindamba Primary School, 2016

**Figure 2:**
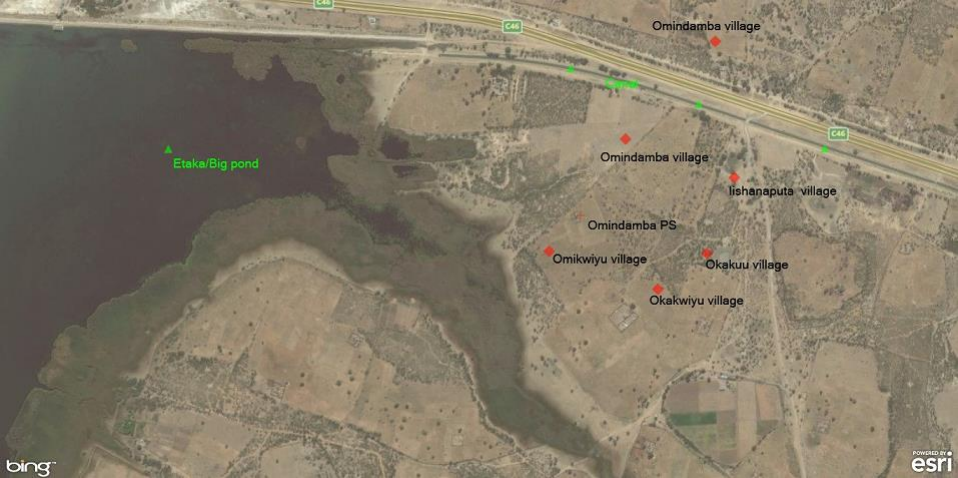
A satellite map showing the water canal and villages of cases in Omusati region with Schistosomiasis cases, March 2016

A total of 250 students were recruited for the case-control study. There were 75 (60%) males and 50 (40%) females, in both cases and controls. The mean age for cases was 11.3 years and controls was 11.0 years, while the standard deviation was 3.2 years in both cases and controls. The most affected age group was 11-15 years with 63(50.4%) cases, followed by 6-10 years with 51 (40.8%) cases. Students were from the five villages surrounding the school, namely; Okakuu, Okakwiyu, Omikwiyu, Omindamba, and Iishanaputa. The majority of the cases were from Omindamba village 57/125 (45.6%), followed by Omikwiyu village 35/125 (28%). Most cases and controls depended on ponds and canal water for drinking and other domestic uses. Of total cases and controls; 121 (96.8%) and 110 (88.0%) reported that they swam in canal water (Table 1). The canal in the region runs through Omindamba village which had the majority of cases, 57 (45.6%). Omikwiyu village, which had the second highest number of cases among students 35 (28.0%), is closer to Etaka pond, compared to other villages. Iishanaputa with 1 (0.8%) case is far from Etaka pond but closer to the canal (Figure 1, Figure 2).

**Table 1:** Socio-demographic characteristics of cases and controls of Omindamba primary school, 2016

Characteristics	Cases		Control	
	n=125 (%)		n=125 (%)	
**Gender**				
Female	50	(40)	50	(40)
Male	75	(60)	75	(60)
**Age**				
Mean (years)	11.3	-	11.0	-
Standard Deviation (years)	3.2	-	3.2	-
6-10 years	51	(40.8)	56	(44.8)
11-15 years	63	(50.4)	59	(47.2)
16-19 years	11	(8.8)	10	(8)
**Village**				
Iishanaputa	1	(0.8)	0	(0.0)
Okakwiyu	13	(10.4)	3	(2.4)
Okakuu	19	(15.2)	33	(26.6)
Omikwiyu	35	(28.0)	25	(20.2)
Omindamba	57	(45.6)	63	(50.8)
**Non-employed caregivers**				
Yes	34	(27.2)	45	(36.0)
No	91	(72.8)	80	(64.0)
**Source of drinking water**				
Tap water				
Yes	31	(24.8)	43	(34.4)
No	94	(75.2)	82	(65.6)
Canal				
Yes	33	(26.4)	45	(36.0)
No	92	(73.6)	80	(64.0)
Pond-1				
Yes	43	(34.4)	32	(25.6)
No	82	(65.6)	93	(74.4)
Pond-2/ Etaka				
Yes	42	(33.6)	24	(19.2)
No	83	(66.4)	101	(80.8)
**Swimming in Canal**				
Yes	121	(96.8)	110	(88.0)
No	4	(3.2)	15	(12.0)

Male students were more affected, but there was no gender association (odds ratio OR =1.0; 95% CI=0.6-1.7; p-value=0.9). There was no association between using a tap (OR = 0.63; 95% CI = 0.4-1.1; p-value = 0.13) or a canal OR = 0.63; CI = 0.4-1.1; p-value = 0.13) as a source of drinking water with being a case. Swimming in the canal (OR = 4.13; 95% CI: 1.3-12.8; p-value = 0.02) was significantly associated with the infection. Having none of the caregivers employed (OR = 1.52, 95% CI: 0.9-2.6, p-value = 0.07) was not associated with being a case (Table 2). The independent factors for being a case for dysuria and haematuria were swimming in the canal (adjusted OR = 3.58; 95% CI = 1.14-11.2; p-value = 0.03) and using Etaka pond as a source of water (adjusted OR = 1.95; 95% CI = 1.09-3.50, p-value = 0.02) from an unconditional logistic regression model (Table 3).

**Table 2:** Factors associated with dysuria and haematuria among students at Omindamba Primary School, 2016

Exposure	Cases	Controls	OR	95% CI	P-value
	n=125 (%)	N=125 (%)			
**Gender**					
Female	50 (40.0)	50 (40.0)	1.0	(0.6-1.7)	0.9
Male	75 (60.0)	75 (60.0)			
**Source of drinking water**					
Tap water					
Yes	31 (24.8)	43 (34.4)	0.63	(0.4-1.1)	0.13
No	94 (75.2)	82 (65.6)			
Canal					
Yes	33 (26.4)	45 (36.0)	0.64	(0.4-1.1)	0.13
No	92 (73.6)	80 (64.0)			
Pond-1					
Yes	43 (34.4)	32 (25.6)	1.52	(0.9-2.6)	0.13
No	82 (65.6)	93 (74.4)			
Pond-2/ Etaka					
Yes	42 (33.6)	24 (19.2)	2.13	(1.2-3.8)	0.01+
No	83 (66.4)	101 (80.8)			
**Swimming in canal**					
Yes	121 (96.8)	110 (88.0)	4.13	(1.3-12.8)	0.02+
No	4 (3.2)	15 (12.0)			
**Boil ground water**					
Yes	7 (6.1)	10 (9.3)	0.64	(0.2-1.7)	0.52
No	108 (93.9)	98 (90.7)			
**Non-employed caregiver**					
Yes	34 (27.2)	45 (36.0)	1.52	(0.9-2.6)	0.07
No	91 (72.8)				

**Table 3:** Independent predictors of being a case of dysuria and haematuria based on unconditional logistic regression at Omindamba School, 2016

Variables	Adjusted OR	95% CI	P-value
Swimming in the canal	3.58	1.14-11.2	0.03
Etaka (big pond) as source of water	1.95	1.09-3.50	0.02

## Discussion

In our investigation, we found that the cause of dysuria and haematuria among students of Omindamba primary school was *Schistosoma hematobium* infection. From our case-control study we found that swimming in the canal, and using water from the Etaka- pond were the independent factors for being a case for dysuria and haematuria. These findings are similar to a study done in Zimbabwe, where there were significant associations between having schistosomiasis infection and participating in swimming, bathing in the dam, fishing using a fishing line and fishing with legs in water [13]. However, drinking from the canal was not found to be significantly associated with Schistosomiasis in our study because only 33 (26.4%) of cases drank from the canal as opposed to 121 (96.8%) of cases who swam in the Canal. A systematic review showed that access to safe water supplies was found to be associated with significantly less likelihood of infection with *S. haematobium, S. mansoni, and S. japonicum* [14]. In our study, a higher percentage of the controls compared to cases use tape water, while less percentage of the controls swam in the canal. Secondly, a higher percentage of the controls boiled the ground water compared to the cases. This is a clearly shows that the cases were more predisposed to the risk of being infected.

We also found that more males were infected compared to females with no significant association. Socio-cultural factors, where males are mostly engaged in water-contact activities like swimming and bathing, fishing, farming and watering cattle could lead to higher exposure among males. This finding is consistent with studies conducted in Ethiopia, Nigeria, Malawi, and Namibia [15-18]. On the contrary, studies carried out in Zimbabwe, Ghana and Nigeria showed a higher prevalence of urinary schistosomiasis among females than males [13,19,20]. This discrepancy with our findings could be due to the differences in cultural practices that expose females to Schistosomiasis risks.

While some of the students who had schistosomiasis also had access to safe running water, it seems that the ability of safe water sources to prevent *Schistosoma* infection would depend on how well they prevent dermal contact with schistosome-infested environmental water bodies. In the case of this outbreak, there might be a lack of knowledge about schistosomiasis among students and their parents because a study conducted by Uusiku and Small, (2009) in the same region in Namibia indicated a low level of knowledge among school children [18]. This finding is consistent with a study done in Mozambique which revealed that though awareness on schistosomiasis was high, knowledge of how it is acquired, transmitted, and prevented was low among those who have heard of the disease [21]. It also points out that, misconceptions, such as the belief that schistosomiasis is transmitted through sexual contact were common [21].

Our study found students who had no employed caregiver at home more at risk of contracting the infection compared to those who had at least one employed caregiver. However, this association was not statistically significant. Employed individuals are likely to have safe running water supplies in their homes. Hence, students with no employed caregiver can easily be exposed to infested sources when fetching water for domestic uses. This finding is comparable to a study from China, where unemployed individuals had a high prevalence of schistosomiasis [22]. An earlier study by Uusiku and Small (2009) in the same region as the current investigation revealed that most of the cases had at least one caregiver working; in comparison to those who had no working caregiver [18].

There were at least three limitations to the generalization of the findings of this study. Firstly, the results of this study cannot be generalized to the population in Omusati region as the study focused on students of a specified school only, and from few villages of the region. Secondly, some controls could have been latent cases as a result of incubation period during the time of the study and therefore will not present with haematuria according the case-definition and hence will underestimate the effect that is, biasing the true association toward the null. Lastly, the young age of students may have limited some necessary information, e.g., the educational and employment status of the caretakers; that could be possible contributing factors to *Schistosoma* infections.

## Conclusion

In conclusion, there was a *Schistosomiasis* outbreak among pupils attending at Omindamba primary school which was caused by *Schistosoma hematobium*. Swimming in the canal and using Etaka pond as a source of drinking water were the predictive factors for the infection. The outbreak among school students presents a high risk of urinary schistosomiasis within the community around Omindamba primary school and the canal. Our study indicates that Schistosomiasis is likely to be governed by social and economic contexts. The disease is closely linked with lack of essential recreational and social services such as free-swimming pools with clean water, and proper sanitation. Information on the transmission and prevention of Schistosomiasis in Omusati region is fragmented. A study to assess the knowledge, attitude and practice toward Schistosomiasis needs to be done.

### What is known about this topic

- Schistosomiasis infection is endemic in most part of sub-Saharan Africa;
- Coming into contact with infected water bodies causes infection;
- Most cases of Schistosomiasis (bloody urine) are unreported as it is seen as usual part of adolescent life in the endemic communities.

### What this study adds

- Lack of knowledge on the transmission of Schistosomiasis contributed to the use of the infested water body;
- The study highlighted the importance of health education in the control and prevention of schistosomiasis and the need to provide safe water for consumption and alternative recreational facilities especially for swimming.

## Competing interests

The authors declare no competing interests.
